# Cefaclor causes vagus nerve-mediated depression-like symptoms with gut dysbiosis in mice

**DOI:** 10.1038/s41598-023-42690-1

**Published:** 2023-09-19

**Authors:** Min-Kyung Joo, Yoon-Jung Shin, Dong-Hyun Kim

**Affiliations:** Neurobiota Research Center and Department of Biomedical and Pharmaceutical Sciences, College of Pharmacy, Kyung Hee University, Seoul, 02447 Korea

**Keywords:** Microbiology, Risk factors, Drug safety

## Abstract

Antibiotics are increasingly recognized as causing neuropsychiatric side effects including depression and anxiety. Alterations in central serotonin and 5-HT receptor expression are implicated in the pathogenesis of anxiety and depression, which are highly comorbid with gastrointestinal disorders. Nevertheless, it is still unclear how antibiotics can cause anxiety and depression. In this study, oral administration of cefaclor, a second-generation cephalosporin antibiotic, induced anxiety- and depression-like behaviors and colitis with gut microbiota alteration in mice. Cefaclor reduced serotonin levels and fluctuated 5-HT receptor mRNA expressions such as *Htr1a*, *Htr1b*, and *Htr6* in the hippocampus. Vagotomy attenuated the cefaclor-induced anxiety- and depression-like symptoms, while the cefaclor-induced changes in gut bacteria alteration and colitis were not affected. Fluoxetine attenuated cefaclor-induced anxiety- and depression-like behaviors. Furthermore, fluoxetine decreased cefaclor-resistant *Enterobacteriaceae* and *Enterococcaceae*. Taken together, our findings suggest that the use of antibiotics, particularly, cefaclor may cause gut dysbiosis-dependent anxiety and depression through the microbiota-gut-blood–brain and microbiota-gut-vagus nerve-brain pathway. Targeting antibiotics-resistant pathogenic bacteria may be a promising therapeutic strategy for the treatment of anxiety and depression.

## Introduction

Depression is one of the most common causes of disability^1^. Worldwide, it is estimated that > 5% of adults suffer from depression^2^. Genetic and environmental factors such as hormone imbalance and medications are involved in the onset of depression^3^. Of these factors, antibiotics such as ampicillin and ciprofloxacin are proposed to increase the risk of depression because they cause gut microbiota dysbiosis^4,5^.

The gut microbiota composition is important in managing mental health^6^. Gut microbiota comprises > 1,000 species from relatively few phyla including Firmicutes, Bacteroidetes, and Proteobacteria^7^. The gut microbiota and bacteria-derived byproducts stimulate the enteric neuroimmune systems in the gastrointestinal tract, which propagate to other organs by regulating the secretion of cytokines and adrenal hormones^8^. Germ-free mice show anxiety-like behavior compared with specific pathogen-free mice^9^. However, fecal microbiota transplantation from specific pathogen-free mice attenuates anxiety in germ-free mice^9^. Recent studies have reported that the transplantation of human fecal microbiota from patients with depression into rodents caused depression-like behavior^10^.

Antibiotics are an effective treatment for bacterial infections, and their use has been associated with the change of gut microbiota composition in humans as well as animals^11–14^. Gut microbiota dysbiosis induced by antibiotics can inhibit the host’s homeostatic functions including the immune, endocrine, and nervous systems, resulting in psychiatric disorders^15–17^. In empirical antibiotic therapy, penicillin-type and cephalosporin-type antibiotics are commonly selected as antimicrobial regimens. These antibiotic treatments can influence mental health problems as adverse effects result from the alteration of gut microbiota^18,19^. For example, researchers have reported that ampicillin-induced gut microbiota dysbiosis causes depression with gut inflammation^20–22^. In addition, combined treatment using amoxicillin and cefotaxime causes an increased abundance of *Klebsiella* spp., pathogenic bacteria, and *Enterococcus* spp., conditional pathogenic bacteria, which are overpopulated in the gut microbiota of patients with depression^23–25^. The proportion of *Enterobacteriaceae* and *Enterococcaceae* increased after oral administration with cefaclor, while *Bifidobacterium* and *Lactobacillus* were reduced after oral administration with cefaclor^26,27^. However, there is little understanding of the effects of orally administered cephalosporin-type antibiotics on gut microbiota dysbiosis and psychiatric disorders.

Neurotransmitters and neurotrophic factors are important for the regulation of the microbiota-gut-brain axis^4^. Many researchers have reported that neuronal factors including serotonin, γ-aminobutyric acid (GABA), and brain-derived neurotrophic factor (BDNF) can be modulated by intestinal microflora, such as *Enterococcus*, *Lactobacillus*, *Escherichia*, and *Bifidobacterium*^20,22,28^. These gut bacteria are considered to play critical roles in the pathogenesis of depression. Studies have shown that serotonin levels in the hippocampus were reduced and the conversion of HIAA/5-HT increased after antibiotics treatment, indicating that serotonin is associated with antibiotics-induced depression-like behaviors in rats^29^. Antibiotics such as cefaclor as major disruptors of gut microbiota might lead to altered gut microbiota and imbalance of neurotransmitter expression, which consequently increase the risk of depression.

Antibiotics induce gut dysbiosis, and their associated side effects include anxiety and depression^4^. We hypothesized that cefaclor could increase the risk for depression due to the alteration of gut microbiota via dysregulation of neurotransmitters in the brain. To explore the role of antibiotics on the occurrence of anxiety and depression, we orally gavaged cefaclor in mice and examined depression-like behaviors and its related biomarkers. Cefaclor caused gut dysbiosis and affected the expression of serotonin and 5-HT receptor in the hippocampus. Celiac vagotomy significantly attenuated cefaclor-induced anxiety- and depression-like behaviors and symptoms in mice. Fluoxetine treatment rescued cefaclor-induced anxiety- and depression-like behaviors in mice with or without celiac vagotomy.

## Results

### Behavioral effect of cefaclor administration

**Figure 1:**
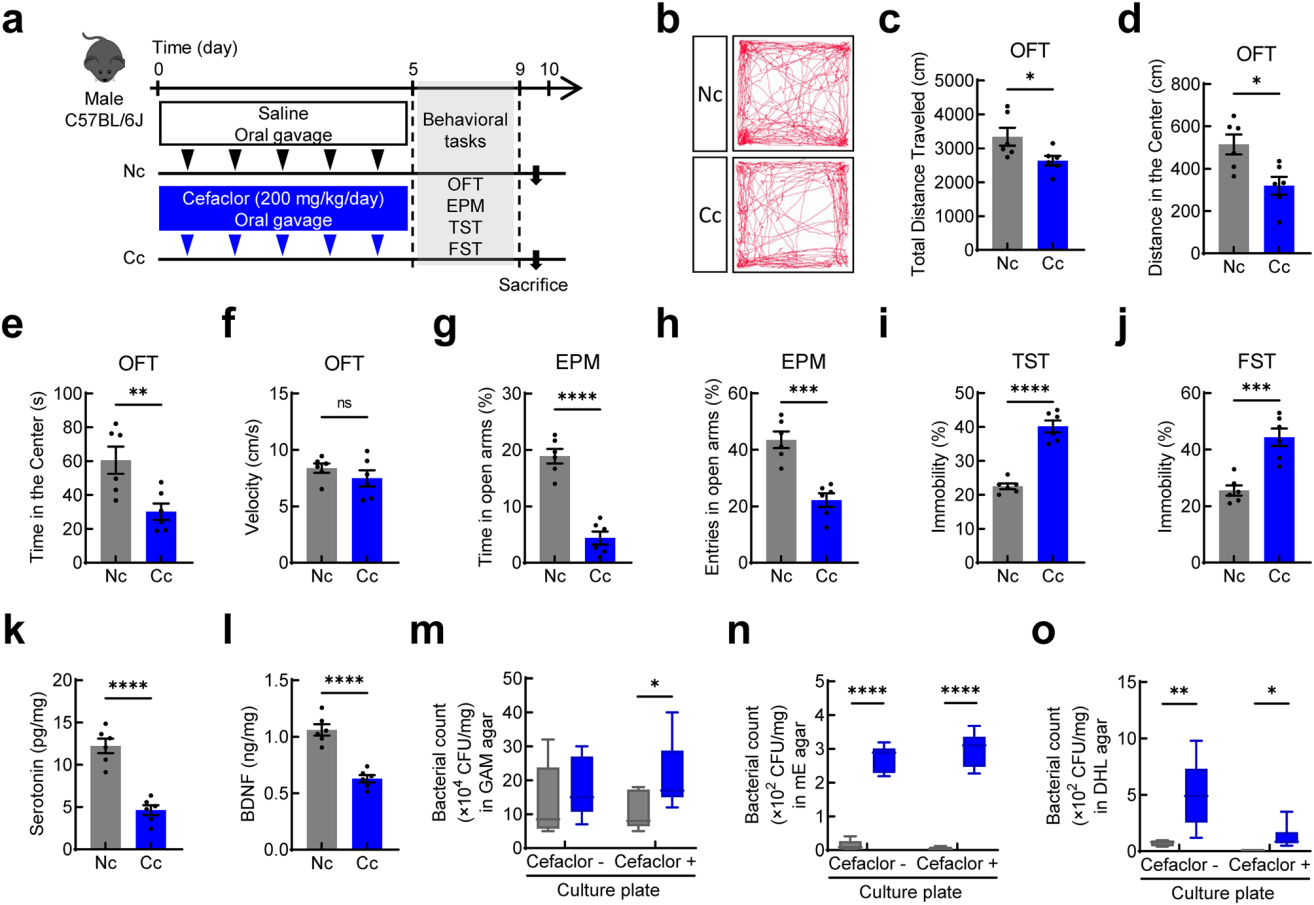
Effects of orally administered cefaclor on anxiety- and depression-like behaviors and gut microbiota in mice. (**a**) A Schematic diagram of the study. The effect of cefaclor on track path (**b**), total distance traveled (**c**), distance in the center (**d**), time in the center (**e**), and velocity (**f**) in OFT. Time spent in open arms (**g**) and entries in open arms (**h**) in EPM. Time spent immobile (**i**) in TST. Time spent immobile in FST (**j**). Serotonin levels (**k**), and BDNF levels (**l**) in the hippocampus. Bacterial colony forming unit on GAM ager plate (**m**), mE agar plate (**n**), and DHL agar plate (**o**) in the colon contents. Control group, dark gray bar; cefaclor-treated group, blue bar. Data are represented as mean ± S.E.M (n = 6/group). Statistical significance between two groups was calculated using two-tailed unpaired *t* test (*p < 0.05, **p < 0.01, ***p < 0.001, ****p < 0.0001).

To investigate the effect of gut microbiota disruptors on anxiety- and depression-like behaviors, mice were orally administered cefaclor once a day for 5 days and behavioral tests relevant to anxiety and depression were carried out (Fig. 1a). Cefaclor administration indued anxiety-like behaviors in mice. In the OFT, the total distance traveled (*t* = 2.361, df = 10, *p* = 0.0399; Fig. 1b,c) decreased in mice treated with cefaclor compared with control mice. The distance in the center (*t* = 3.079, df = 10, *p* = 0.0117; Fig. 1d) and time spent in the center (*t* = 3.236, df = 10, *p* = 0.0089; Fig. 1e) decreased, while there was no significant difference in velocity (*t* = 1.090, df = 10, *p* = 0.3015; Fig. 1f). In the EPM, the time spent in open arms (*t* = 8.259, df = 10, *p* < 0.0001; Fig. 1g) and entries in open arms (*t* = 5.614, df = 10, *p* = 0.0002; Fig. 1h) decreased in mice treated with cefaclor compared with control mice. Regarding depression-related behaviors, the tail suspension test (TST) and forced swim test (FST) analysis showed that the time spent immobile increased in mice treated with cefaclor compared with control mice (*t* = 8.971, df = 10, *p* < 0.0001; Fig. 1i and t = 5.205, df = 10, *p* = 0.0004; Fig. 1j).

### Effect of cefaclor on serotonin and BDNF levels in the hippocampus

Exposure to cefaclor significantly decreased serotonin levels in the hippocampus (*t* = 7.318, df = 10, *p* < 0.0001; Fig. 1k). The levels of BDNF were significantly lower in mice treated with cefaclor than the levels of the neurotropic factor in control mice (*t* = 7.208, df = 10, *p* < 0.0001; Fig. 1l).

### Effect of cefaclor on gut microbiota composition

To investigate changes in fecal bacterial composition after cefaclor treatment, the resistant bacteria and opportunistic pathogens were detected by culture counting. No significant difference in the number of bacterial colonies was observed between control mice and cefaclor-treated mice in a GAM agar plate without antibiotics, whereas it increased in a GAM agar plate with antibiotics in cefaclor-treated mice (control plate: *t* = 0.7060, df = 10, *p* < 0.4963, and cefaclor plate: *t* = 2.253, df = 10, *p* = 0.0479; Fig. 1m). The number of bacterial colonies increased between control mice and cefaclor-treated mice both in a mE agar plate with antibiotics and without antibiotics (control plate: *t* = 15.05, df = 10, *p* < 0.0001, and cefaclor plate: *t* = 13.89, df = 10, *p* < 0.0001; Fig. 1n). In a DHL agar plate, an increased number of bacterial colonies was observed in cefaclor-treated mice (control plate: *t* = 3.549, df = 10, *p* = 0.0053, and cefaclor plate: *t* = 2.792, df = 10, *p* = 0.0190; Fig. 1o).

### Effect of cefaclor on anxiety- and depression-like behaviors in vagotomized mice

**Figure 2:**
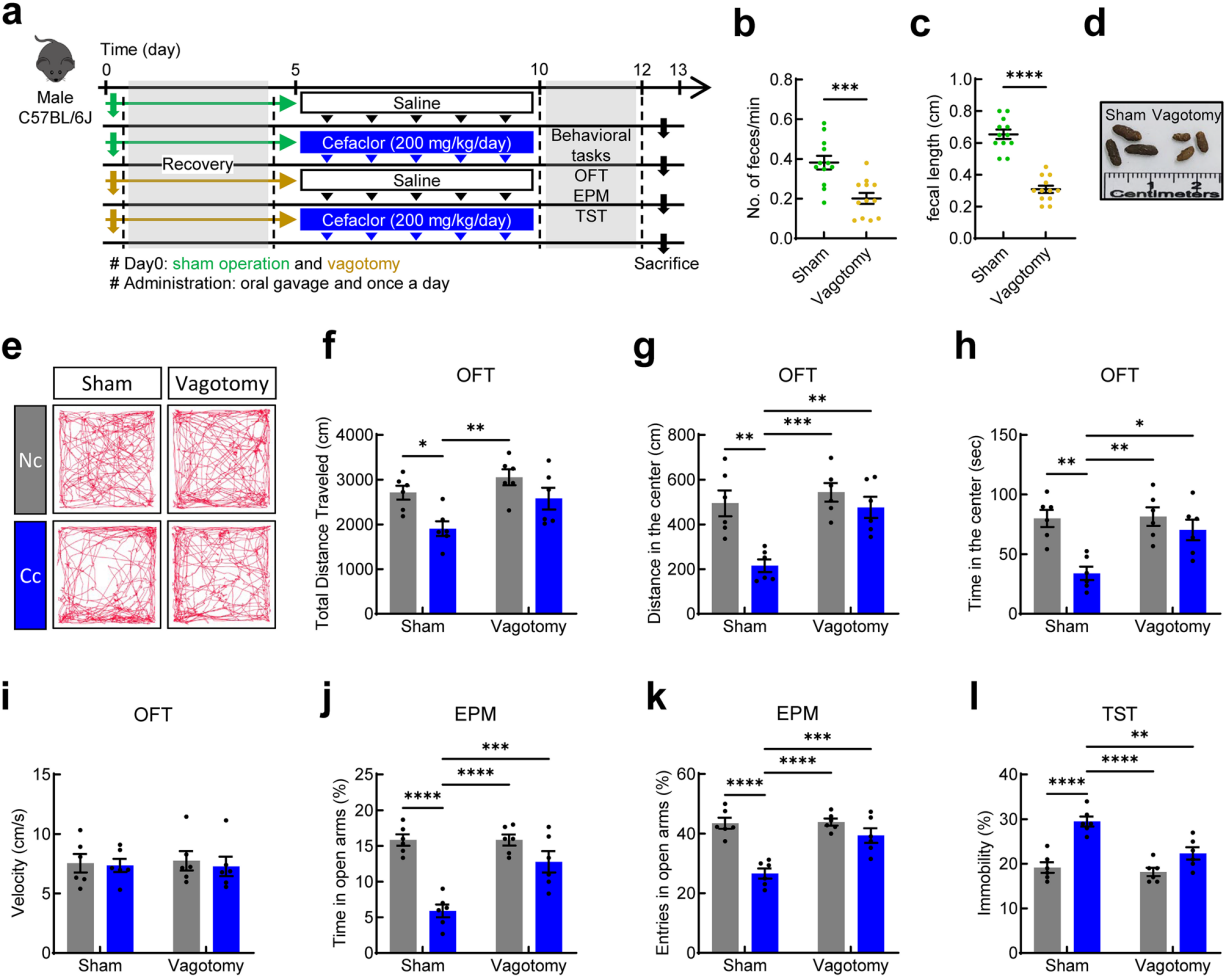
Effect of orally administered cefaclor on anxiety- and depression-like behaviors in vagotomized mice. (**a**) A Schematic diagram of the study. The number of feces per minute (**b**), fecal length (**c**), and fecal color (**d**) between sham-operated mice and vagotomized mice. The effect of cefaclor on track path (**e**), total distance traveled (**f**), distance in the center (**g**), time in the center (**h**), and velocity (**i**) in OFT. Time spent in open arms (**j**) and entries in open arms (**k**) in EPM. Time spent immobile (**l**) in TST. Control group, dark gray bar; cefaclor-treated group, blue bar. Data are represented as mean ± S.E.M (n = 6/group). Statistical significance was calculated using a two-way ANOVA with post-hoc Tukey’s multiple comparisons tests (*p < 0.05, **p < 0.01, ***p < 0.001, ****p < 0.0001).

To further understand the role of the vagus nerve in the cefaclor-induced anxiety and depression, celiac vagotomy (Vx) was carried out, and behavioral changes were measured by using EPM and TST (Fig. 2a). After the operation, vagotomy was validated by measuring fecal pellet parameters. In vagotomized mice, the number of fecal pellets per minute decreased (*t* = 4.119, df = 22, *p* = 0.0005; Fig. 2b). It was observed with lower fecal length (*t* = 9.328, df = 22, *p* < 0.0001; Fig. 2c), and lighter brown color (Fig. 2d) in vagotomized mice compared with sham-operated mice. Validated mice were used in the next experiments. In the OFT, EPM and TST, two-way ANOVA revealed that there was an overall effect of Vx, an overall effect of cefaclor administration, and an interaction between Vx and cefaclor.

In the OFT, the total distance traveled [effect of Vx: *F* (1, 20) = 7.275, *p* = 0.0139; effect of cefaclor: *F* (1, 20) = 11.46, *p* = 0.0029; interaction between Vx and cefaclor: *F* (1, 20) = 0.7465, *p* = 0.3978; Fig. 2e,f] decreased in mice treated with cefaclor compared with control mice. The distance in the center [effect of Vx: *F* (1, 20) = 11.95, *p* = 0.0025; effect of cefaclor: *F* (1, 20) = 14.83, *p* = 0.0010; interaction between Vx and cefaclor: *F* (1, 20) = 5.601, *p* = 0.0282; Fig. 2g] and time spent in the center [effect of Vx: *F* (1, 20) = 6.601, *p* = 0.0183; effect of cefaclor: *F* (1, 20) = 15.02, *p* = 0.0009; interaction between Vx and cefaclor: *F* (1, 20) = 5.585, *p* = 0.0284; Fig. 2h] decreased, while there was no significant difference in velocity [effect of Vx: *F* (1, 20) = 0.0059, *p* = 0.9395; effect of cefaclor: *F* (1, 20) = 0.1925, *p* = 0.6656; interaction between Vx and cefaclor: *F* (1, 20) = 0.03866, *p* = 0.8461; Fig. 2i].

In the EPM, Vx prevented the anxiety-like behaviors induced by cefaclor, which was reflected in having no significant difference in time spent in open arms [effect of Vx: *F* (1, 20) = 11.10, *p* = 0.0033; effect of cefaclor: *F* (1, 20) = 39.52, *p* < 0.0001; interaction between Vx and cefaclor: *F* (1, 20) = 11.09, *p* = 0.0033; Fig. 2j] in vagotomized mice (*p* > 0.05), and entries in open arms [effect of Vx: *F* (1, 20) = 12.61, *p* = 0.0020; effect of cefaclor: *F* (1, 20) = 33.54, *p* < 0.0001; interaction between Vx and cefaclor: *F* (1, 20) = 11.13, *p* = 0.0033; Fig. 2k] in vagotomized mice (*p* > 0.05). In the TST, Vx prevented the depression-like behaviors induced by cefaclor. No significant differences were observed in time spent immobile [effect of Vx: *F* (1, 20) = 12.31, *p* = 0.0022; effect of cefaclor: *F* (1, 20) = 38.82, *p* < 0.0001; interaction between Vx and cefaclor: *F* (1, 20) = 7.021, *p* = 0.0154; Fig. 2l] in vagotomized mice (*p* > 0.05).

### Effect of cefaclor on serotonin-involved signals in the hippocampus of vagotomized mice

**Figure 3:**
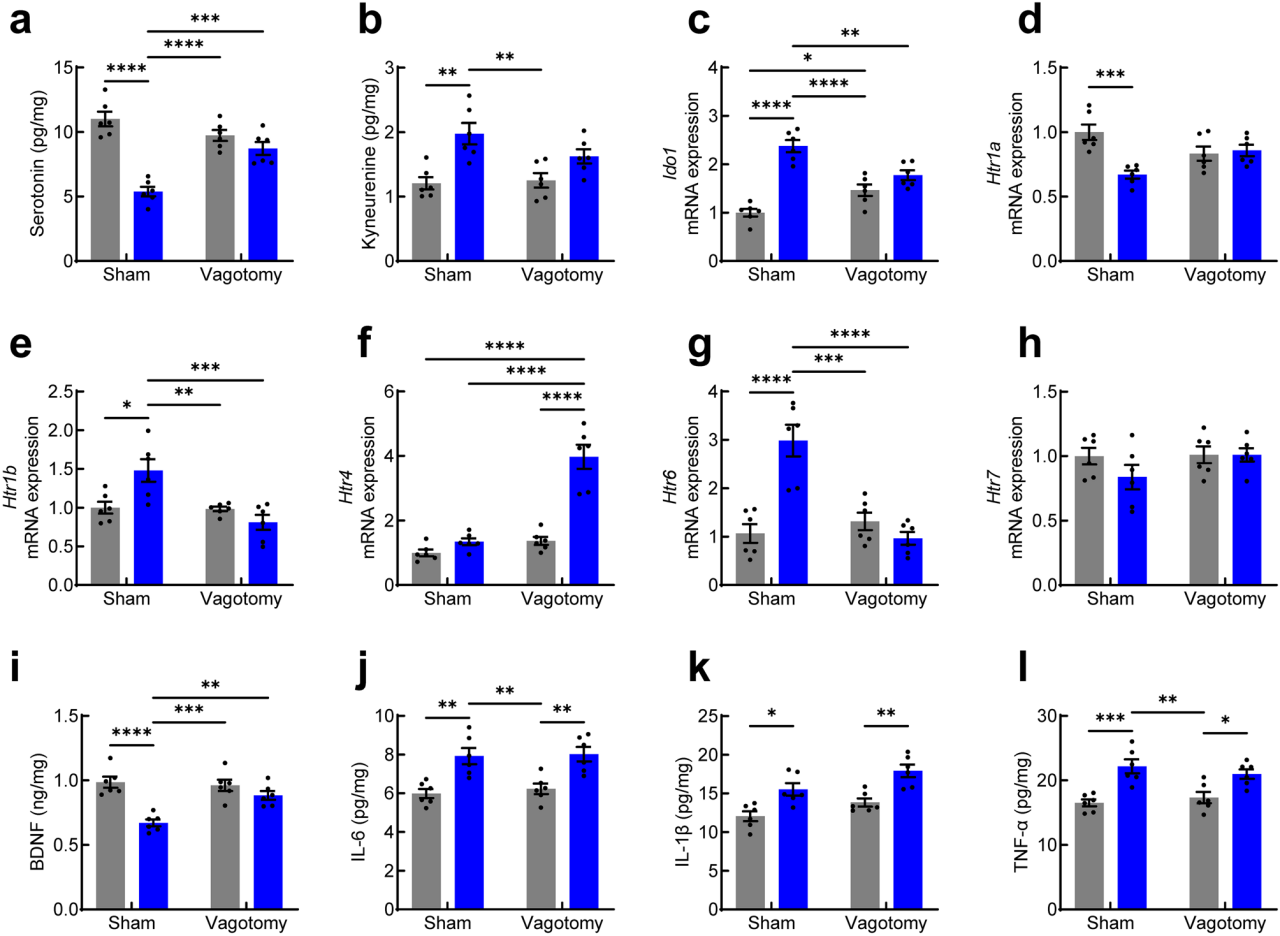
Effect of orally administered cefaclor on the expression of serotonin, 5-HT receptor, and pro-inflammatory cytokine in the hippocampus of vagotomized mice. The expression of serotonin (**a**), and kynurenine (**b**) in the hippocampus. The expression of *Ido1* (**c**), *Htr1a* (**d**), *Htr1b* (**e**), *Htr4* (**f**), *Htr6* (**g**), and *Htr7* (**h**) mRNA in the hippocampus. The expression of BDNF (**i**), IL-6 (**j**), IL-1β (**k**), and TNF-α (**l**) in the hippocampus. Control group, dark gray bar; cefaclor-treated group, blue bar. Data are represented as mean ± S.E.M (n = 6/group). Statistical significance was calculated using a two-way ANOVA with post-hoc Tukey’s multiple comparisons tests (*p < 0.05, **p < 0.01, ***p < 0.001, ****p < 0.0001).

To explore the effects of cefaclor on serotonin-associated signals in the hippocampus, we examined how cefaclor can affect serotonin and 5-HT receptor mRNA expression. We observed the effects of vagotomy on the change of serotonin-associated signals in the hippocampus of mice treated with cefaclor. Statistical analysis revealed that there was a significant interaction between cefaclor treatment and Vx in the levels of serotonin in the hippocampus [effect of Vx: *F* (1, 20) = 4.826, *p* = 0.0400; effect of cefaclor: *F* (1, 20) = 49.60, *p* < 0.0001; interaction between Vx and cefaclor: *F* (1, 20) = 23.98, *p* < 0.0001; Fig. 3a], whereas the levels of kynurenine had no interaction between cefaclor treatment and Vx [effect of Vx: *F* (1, 20) = 1.533, *p* = 0.2300; effect of cefaclor: *F* (1, 20) = 21.25, *p* = 0.0002; interaction between Vx and cefaclor: *F* (1, 20) = 2.562, *p* = 0.1251; Fig. 3b]. Post hoc analysis found that in sham-operated mice, cefaclor reduced the levels of serotonin in the hippocampus (*p* = 0.0031), which is consistent with our initial findings (Fig. 1k). This effect on serotonin levels was prevented by Vx. Regarding mRNA expressions, exposure to cefaclor affected the expressions of *Ido1*, *Htr1a*, *Htr1b*, *Htr4*, and *Htr6* in the hippocampus between sham-operated mice and vagotomized mice. There was a significant interaction between cefaclor treatment and Vx on the expressions of *Ido1* mRNA [effect of Vx: *F* (1, 20) = 0.3808, *p* = 0.5441; effect of cefaclor: *F* (1, 20) = 60.94, *p* < 0.0001; interaction between Vx and cefaclor: *F* (1, 20) = 24.25, *p* < 0.0001; Fig. 3c], *Htr1a* mRNA [effect of Vx: *F* (1, 20) = 0.04769, *p* = 0.8293; effect of cefaclor: *F* (1, 20) = 6.689, *p* = 0.0055; interaction between Vx and cefaclor: *F* (1, 20) = 13.10, *p* = 0.0017; Fig. 3d], *Htr1b* mRNA [effect of Vx: *F* (1, 20) = 12.68, *p* = 0.0020; effect of cefaclor: *F* (1, 20) = 2.519, *p* = 0.1282; interaction between Vx and cefaclor: *F* (1, 20) = 11.50, *p* = 0.0029; Fig. 3e], *Htr4* mRNA [effect of Vx: *F* (1, 20) = 51.32, *p* < 0.0001; effect of cefaclor: *F* (1, 20) = 49.66, *p* < 0.0001; interaction between Vx and cefaclor: *F* (1, 20) = 29.12, *p* < 0.0001; Fig. 3f], and *Htr6* mRNA [effect of Vx: *F* (1, 20) = 15.95, *p* = 0.0007; effect of cefaclor: *F* (1, 20) = 12.57, *p* < 0.0001; interaction between Vx and cefaclor: *F* (1, 20) = 26.26, *p* = 0.0020; Fig. 3g] mRNA in the hippocampus. In sham-operated mice, cefaclor significantly affected the expressions of *Ido1* (*p* < 0.0001), *Htr1a* (*p* = 0.0006), *Htr1b* (*p* = 0.0106), and *Htr6* (*p* < 0.0001) mRNA in the hippocampus in comparison with controls, while it did not in vagotomized mice. The levels of the expressions of *Ido1* mRNA increased in the hippocampus of saline-fed vagotomized mice compared with sham controls (*p* = 0.0299). The levels of *Htr4* mRNA increased in vagotomized mice treated with cefaclor compared with Vx controls (*p* < 0.0001), while they did not in sham-operated mice. There was no interaction between Vx and cefaclor in the levels of *Htr7* mRNA in the hippocampus [effect of Vx: *F* (1, 20) = 1.687, *p* = 0.2617; effect of cefaclor: *F* (1, 20) = 1.334, *p* = 0.2617; interaction between Vx and cefaclor: *F* (1, 20) = 1.322, *p* = 0.2637; Fig. 3h]. Moreover, there was a significant interaction between cefaclor treatment and Vx in the levels of BDNF in the hippocampus [effect of Vx: *F* (1, 20) = 6.327, *p* = 0.0205; effect of cefaclor: *F* (1, 20) = 27.42, *p* < 0.0001; interaction between Vx and cefaclor: *F* (1, 20) = 10.01, *p* = 0.0049; Fig. 3i]. In sham-operated mice, cefaclor reduced the levels of BDNF in the hippocampus (*p* = 0.0031), which is consistent with our initial findings (Fig. 1l). An effect of Vx was observed and the BDNF levels were prevented by Vx. In regard to pro-inflammatory cytokines, the levels of IL-6 [effect of Vx: *F* (1, 20) = 0.2611, *p* = 0.6149; effect of cefaclor: *F* (1, 20) = 30.29, *p* < 0.0001; interaction between Vx and cefaclor: *F* (1, 20) = 0.04906, *p* = 0.8269; Fig. 3j], IL-1β [effect of Vx: *F* (1, 20) = 8.840, *p* = 0.0075; effect of cefaclor: *F* (1, 20) = 29.04, *p* < 0.0001; interaction between Vx and cefaclor: *F* (1, 20) = 0.1970, *p* = 0.6619; Fig. 3k], and TNF-α [effect of Vx: *F* (1, 20) = 0.05123, *p* = 0.8231; effect of cefaclor: *F* (1, 20) = 31.07, *p* < 0.0001; interaction between Vx and cefaclor: *F* (1, 20) = 1.476, *p* = 0.2385; Fig. 3l] had no interaction between cefaclor treatment and Vx. Cefaclor administration increased the levels of IL-6 (*p* = 0.0029, *p* = 0.0060), IL-1β (*p* = 0.0112, *p* = 0.0027), and TNF-α (*p* = 0.0006, *p* = 0.0277) in the hippocampus of both sham-operated and vagotomized mice compared with controls (sham vs. sham + cefaclor, Vx vs. Vx + cefaclor, *p*-value respectively). In the plasma, statistical analysis showed that the levels of IL-6 [effect of Vx: *F* (1, 20) = 1.416, *p* = 0.2480; effect of cefaclor: *F* (1, 20) = 39.03, *p* < 0.0001; interaction between Vx and cefaclor: *F* (1, 20) = 0.000, *p* > 0.9999; Fig. S1a], IL-1β [effect of Vx: *F* (1, 20) = 3.647, *p* = 0.0706; effect of cefaclor: *F* (1, 20) = 51.51, *p* < 0.0001; interaction between Vx and cefaclor: *F* (1, 20) = 2.052, *p* = 0.1674; S1b], and TNF-α [effect of Vx: *F* (1, 20) = 0.7297, *p* = 0.04031; effect of cefaclor: *F* (1, 20) = 33.52, *p* < 0.0001; interaction between Vx and cefaclor: *F* (1, 20) = 0.7297, *p* = 0.4031; S1c] had no interaction between cefaclor treatment and Vx. An effect of cefaclor was only observed. Cefaclor administration increased the levels of IL-6 (*p* = 0.0014, *p* = 0.0014), IL-1β (*p* = 0.0025, *p* < 0.0001), and TNF-α (*p* = 0.0114, *p* = 0.0007) in the plasma of both sham-operated and vagotomized mice compared with controls (sham vs. sham + cefaclor, Vx vs. Vx + cefaclor, *p*-value respectively).

### Effect of cefaclor on gut inflammation and microbiota composition in vagotomized mice

**Figure 4:**
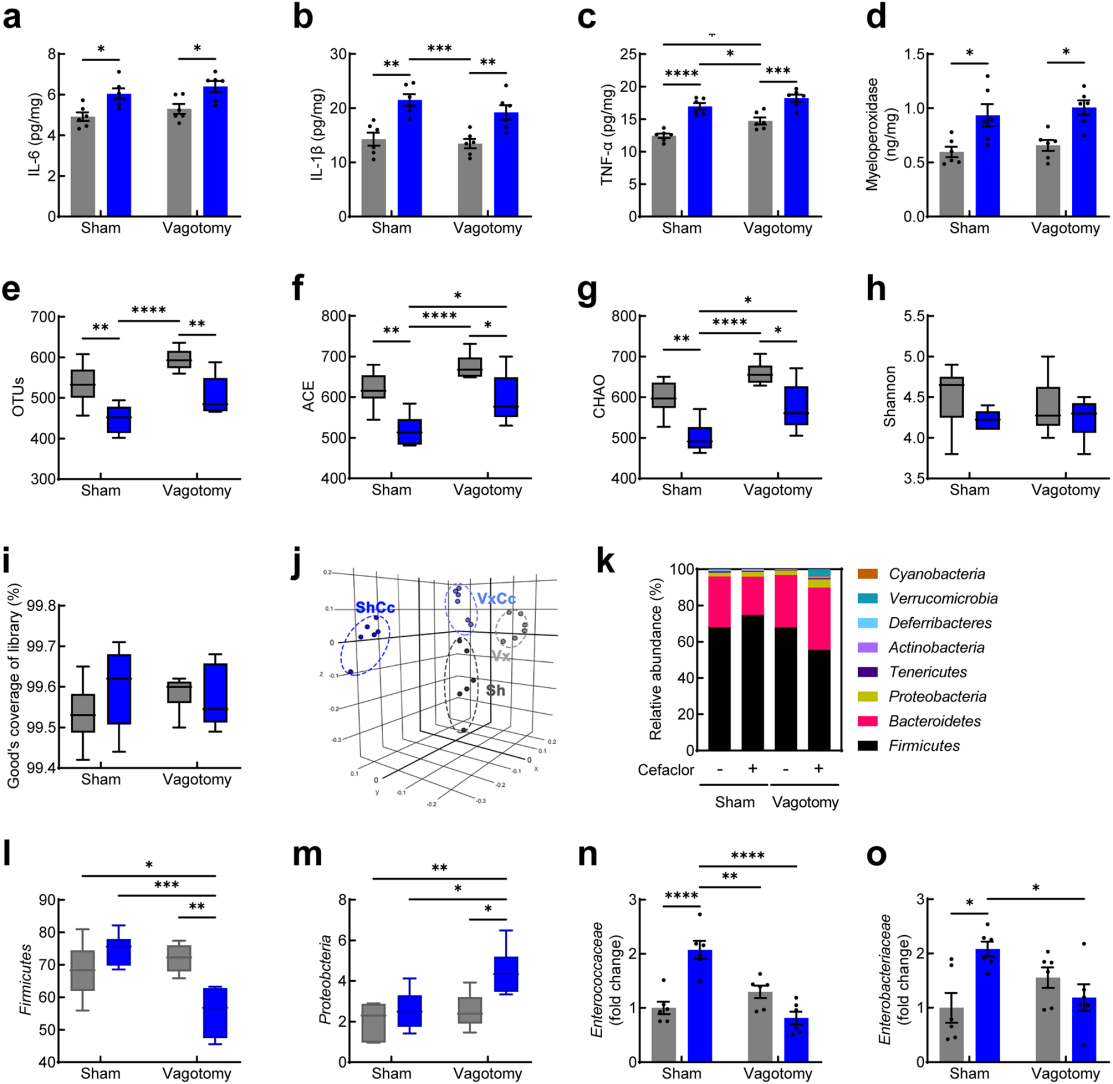
Effect of orally administered cefaclor on colitis and gut microbiota composition in vagotomized mice. The expression of IL-6 (**a**), IL-1β (**b**), TNF-α (**c**), myeloperoxidase (**d**) in the colon. Alpha-diversity OTUs (**e**), ACE (**f**), CHAO (**g**), Shannon (**h**), Good coverage of library (**i**) indexes and beta-diversity (principal component analysis, PCoA) (**j**) and relative abundance of bacterial composition at the phylum level (**k**), the relative abundance of *Firmicutes* (**l**) and *Proteobacteria* (**m**) assessed by pyrosequencing. The relative abundance of *Enterococcaceae* (**n**) and *Enterobacteriaceae* (**o**) in the feces, analyzed by qPCR. Data are represented as mean ± S.E.M (n = 6/group). Statistical significance was calculated using a two-way ANOVA with post-hoc Tukey’s multiple comparisons tests (*p < 0.05, **p < 0.01, ***p < 0.001, ****p < 0.0001).

In the colon, exposure to cefaclor increased pro-inflammatory cytokine and myeloperoxidase expression both in sham-operated and vagotomized mice. Analysis of the levels of IL-6 [effect of Vx: *F* (1, 20) = 2.165, *p* = 0.1568; effect of cefaclor: *F* (1, 20) = 19.73, *p* = 0.0003; interaction between Vx and cefaclor: *F* (1, 20) = 0.001979, *p* = 0.9650; Fig. 4a], IL-1β[effect of Vx: *F* (1, 20) = 1.860, *p* = 0.1877; effect of cefaclor: *F* (1, 20) = 32.89, *p* < 0.0001; interaction between Vx and cefaclor: *F* (1, 20) = 0.4089, *p* = 0.5298; Fig. 4b], TNF-α [effect of Vx: *F* (1, 20) = 12.95, *p* = 0.0018; effect of cefaclor: *F* (1, 20) = 66.28, *p* < 0.0001; interaction between Vx and cefaclor: *F* (1, 20) = 1.090, *p* = 0.3089; Fig. 4c], and myeloperoxidase [effect of Vx: *F* (1, 20) = 0.8756, *p* = 0.3606; effect of cefaclor: *F* (1, 20) = 23.89, *p* < 0.0001; interaction between Vx and cefaclor: *F* (1, 20) = 0.005261, *p* = 0.9429; Fig. 4d] revealed no interaction between cefaclor treatment and Vx, while the effects of cefaclor were observed. Cefaclor administration increased the levels of IL-6 (*p* = 0.0228, *p* = 0.0261), IL-1β (*p* = 0.0011, *p* = 0.0088), TNF-α (*p* < 0.0001, *p* = 0.0004), and myeloperoxidase (*p* = 0.0137, *p* = 0.0109) in the colon of both sham-operated and vagotomized mice compared with controls (sham vs. sham + cefaclor, Vx vs. Vx + cefaclor, *p*-value respectively).

In the gut microbiota, the alteration of microbial composition was observed. There were significant differences in the OTUs [effect of Vx: *F* (1, 20) = 12.07, *p* = 0.0024; effect of cefaclor: *F* (1, 20) = 27.12, *p* < 0.00001; interaction between Vx and cefaclor: *F* (1, 20) = 0.02509, *p* = 0.8757; Fig. 4e], ACE [effect of Vx: *F* (1, 20) = 13.21, *p* = 0.0016; effect of cefaclor: *F* (1, 20) = 24.16, *p* < 0.0001; interaction between Vx and cefaclor: *F* (1, 20) = 0.3220, *p* = 0.5767; Fig. 4f], and CHAO [effect of Vx: *F* (1, 20) = 14.53, *p* = 0.0011; effect of cefaclor: *F* (1, 20) = 26.40, *p* < 0.0001; interaction between Vx and cefaclor: *F* (1, 20) = 0.1524, *p* = 0.7004; Fig. 4g] indexes indicating gut microbial α-diversity, while there was no significant difference in the Shannon [effect of Vx: *F* (1, 20) = 0.2697, *p* = 0.6092; effect of cefaclor: *F* (1, 20) = 3.118, *p* = 0.0927; interaction between Vx and cefaclor: *F* (1, 20) = 0.4327, *p* = 0.5181; Fig. 4h] and Good’s coverage of library [effect of Vx: *F* (1, 20) = 0.1630, *p* = 0.6907; effect of cefaclor: *F* (1, 20) = 0.6961, *p* = 0.4140; interaction between Vx and cefaclor: *F* (1, 20) = 1.600, *p* = 0.2204; Fig. 4i] indexes.

β-diversity based on Bray–Curtis’s dissimilarity between groups showed that the overall structure of the gut microbial community was different (PREMANOVA, *p* = 0.001; Fig. 4j).

At the phylum level, gut microbiota composition was different between the groups (Fig. 4k). Cefaclor administration showed a tendency to increase the relative abundance of *Firmicutes* in sham-operated mice, whereas it significantly decreased the relative abundance of *Firmicutes* in vagotomized mice [effect of Vx: *F* (1, 20) = 8.493, *p* = 0.0086; effect of cefaclor: *F* (1, 20) = 3.507, *p* = 0.0758; interaction between Vx and cefaclor: *F* (1, 20) = 18.48, *p* = 0.0003; Fig. 4l]. Statistical analysis revealed that there was a significant interaction between cefaclor treatment and Vx on the *Firmicutes*. An effect of Vx on the *Firmicutes* was also observed. Cefaclor administration significantly increased the relative abundance of *Proteobacteria* in vagotomized mice [effect of Vx: *F* (1, 20) = 9.262, *p* = 0.0064; effect of cefaclor: *F* (1, 20) = 9.741, *p* = 0.0054; interaction between Vx and cefaclor: *F* (1, 20) = 3.249, *p* = 0.0866; Fig. 4m]. The effects of Vx and cefaclor on the *Firmicutes* were observed. qPCR results showed there was an interaction between Vx and cefaclor in the population of *Enterococcaceae* [effect of Vx: *F* (1, 20) = 13.74, *p* = 0.0014; effect of cefaclor: *F* (1, 20) = 5.156, *p* = 0.0344; interaction between Vx and cefaclor: *F* (1, 20) = 35.90, *p* < 0.0001; Fig. 4n] and *Enterobacteriaceae* [effect of Vx: *F* (1, 20) = 0.6159, *p* = 0.4418; effect of cefaclor: *F* (1, 20) = 2.717, *p* = 0.1149; interaction between Vx and cefaclor: *F* (1, 20) = 11.15, *p* = 0.0033; Fig. 4o]. Cefaclor administration significantly increased the population of *Enterococcaceae* in sham-operated mice. The change of *Enterococcaceae* induced by cefaclor was prevented by Vx.

### Effect of fluoxetine on cefaclor-induced anxiety- and depression-like behaviors, neuroinflammation, gut Inflammation, and fecal microbiota composition in mice

**Figure 5:**
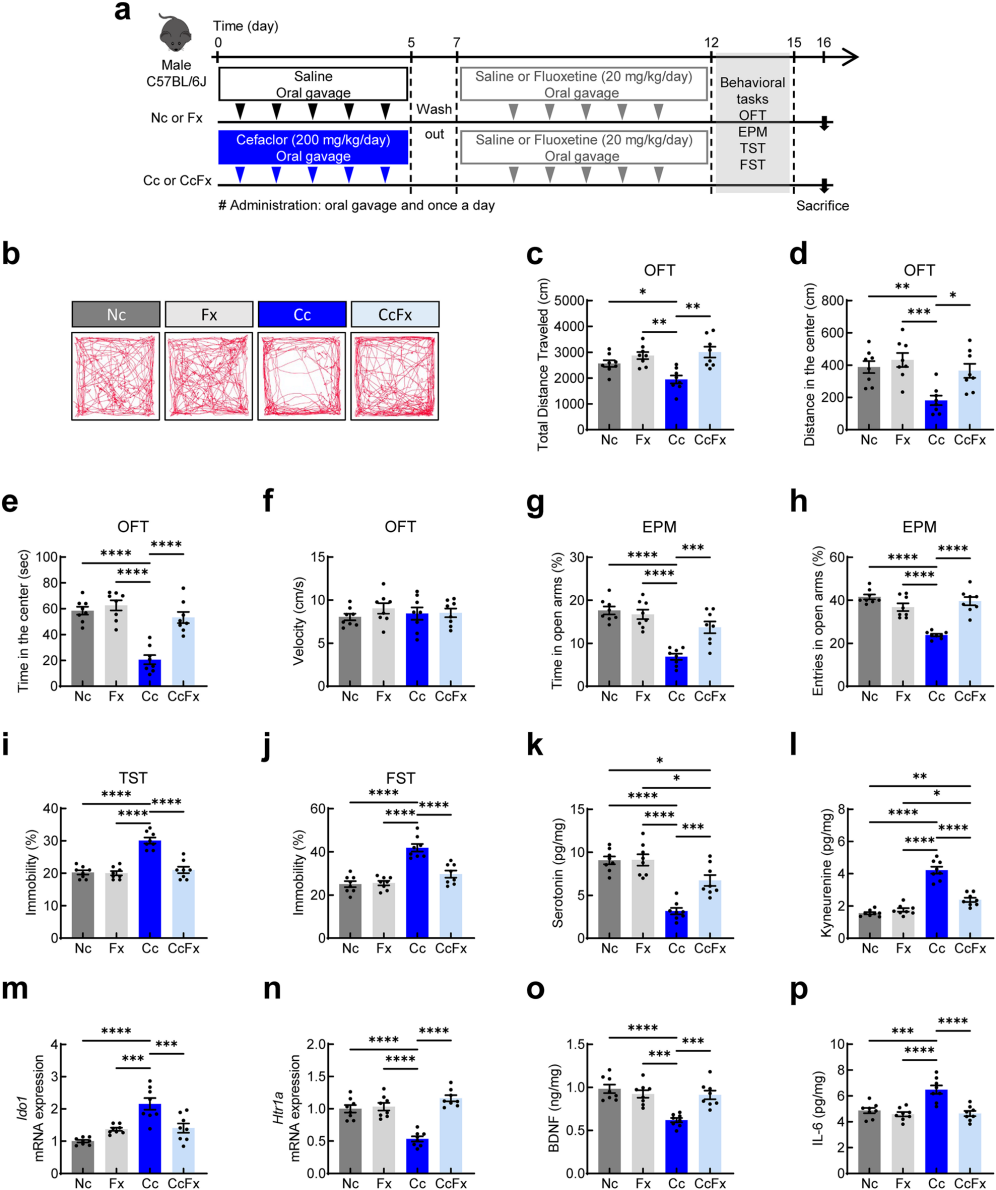
Effect of fluoxetine on anxiety- and depression-like behaviors in cefaclor-induced depressive mice. (**a**) A Schematic diagram of the study. The effect of fluoxetine on track path (**b**), total distance traveled (**c**), distance in the center (**d**), time in the center (**e**), and velocity (**f**) in OFT. Time spent in open arms (**g**) and entries in open arms (**h**) in EPM. Time spent immobile (**i**) in TST. Time spent immobile (**j**) in FST. The effect of fluoxetine on the expression of serotonin (**k**), and kynurenine (**l**) in the hippocampus. The expression of *Ido1* (**m**), *Htr1a* (**n**) mRNA in the hippocampus. The expression of BDNF (**o**), IL-6 (**p**) in the hippocampus. Data are represented as mean ± S.E.M (n = 8/group). Control mice, dark gray bar; mice subjected to fluoxetine alone, light gray bar; mice subjected to cefaclor, blue bar; mice subjected to cefaclor followed by fluoxetine, light blue bar. Statistical significance was calculated using a one-way ANOVA with post-hoc Tukey’s multiple comparisons tests (*p < 0.05, **p < 0.01, ***p < 0.001, ****p < 0.0001).

Fluoxetine is a selective serotonin reuptake inhibitor (SSRI) that is commonly used to treat depression. Fluoxetine increases synaptic serotonin levels in the brain and vagus nerve activity in the gut^30^. Therefore, we next examined the effects of fluoxetine on cefaclor-induced anxiety- and depression-like behaviors in mice (Fig. 5a).

In the OFT, fluoxetine increased the total distance traveled, the distance in the center, and the time spent in the center in mice treated with cefaclor [*F* (3, 28) = 5.396, *p* = 0.0047; Fig. 5b,c, F (3, 28) = 8.135, *p* = 0.0005; Fig. 5d, and F (3, 28) = 26.40, *p* < 0.0001; Fig. 5e]. There was no significant difference between the groups in the velocity [*F* (3, 28) = 0.5250, *p* = 0.6687; Fig. 5f].

In the EPM, fluoxetine increased the time spent in open arms and entries in open arms in mice treated with cefaclor [*F* (3, 28) = 21.28, *p* < 0.0001; Fig. 5g and F (3, 28) = 30.23, *p* < 0.0001; Fig. 5h]. In the TST and FST, fluoxetine treatment decreased immobility in mice treated with cefaclor [*F* (3, 28) = 34.90, *p* < 0.0001; Fig. 5i and F (3, 28) = 27.87, *p* < 0.0001; Fig. 5j].

Fluoxetine increased the serotonin levels [*F* (3, 28) = 26.49, *p* < 0.0001; Fig. 5k] while decreasing the kynurenine levels [*F* (3, 28) = 71.70, *p* < 0.0001; Fig. 5l] in the hippocampus of mice treated with cefaclor. Higher levels of *Ido1* mRNA were founded in the hippocampus of cefaclor-treated mice compared with control mice and fluoxetine (Fx)-treated mice [*F* (3, 28) = 16.36, *p* < 0.0001; Fig. 5m]. Cefaclor decreased the levels of *Htr1a* mRNA, whereas increased the levels of *Htr1b* mRNA in the hippocampus [*F* (3, 28) = 28.60, *p* < 0.0001; Fig. 5n and F (3, 28) = 8.979, *p* = 0.0003; Fig. S2a]. Fluoxetine increased the levels of *Htr4* mRNA in Fx-treated mice compared with control mice and cefaclor-treated mice [*F* (3, 28) = 59.04, *p* < 0.0001; Fig. S2b]. Cefaclor increased the levels of *Htr6* mRNA in the hippocampus of cefaclor-treated mice [*F* (3, 28) = 50.87, *p* < 0.0001; Fig. S2c], while no difference was observed in the levels of *Htr7* mRNA [*F* (3, 28) = 2.539, *p* = 0.0767; Fig. S2d]. Fluoxetine restored the levels of *Htr1a*, *Htr1b*, and *Htr6* mRNA in the hippocampus of cefaclor-treated mice. The BDNF levels decreased by cefaclor and recovered after fluoxetine treatment [*F* (3, 28) = 14.15, *p* < 0.0001; Fig. 5o]. Fluoxetine decreased the elevated levels of IL-6, IL-1β, and TNF-α in the hippocampus [*F* (3, 28) = 15.07, *p* < 0.0001; Fig. 5p, *F* (3, 28) = 25.00, *p* < 0.0001; Fig. S2e, and *F* (3, 28) = 16.39, *p* < 0.0001; Fig. S2f.] and in the plasma of mice treated with cefaclor [*F* (3, 28) = 9.406, *p* = 0.0002; Fig. S3a, *F* (3, 28) = 20.60, *p* < 0.0001; Fig. S3b, and *F* (3, 28) = 15.32, *p* < 0.0001; Fig. S3c].

**Figure 6:**
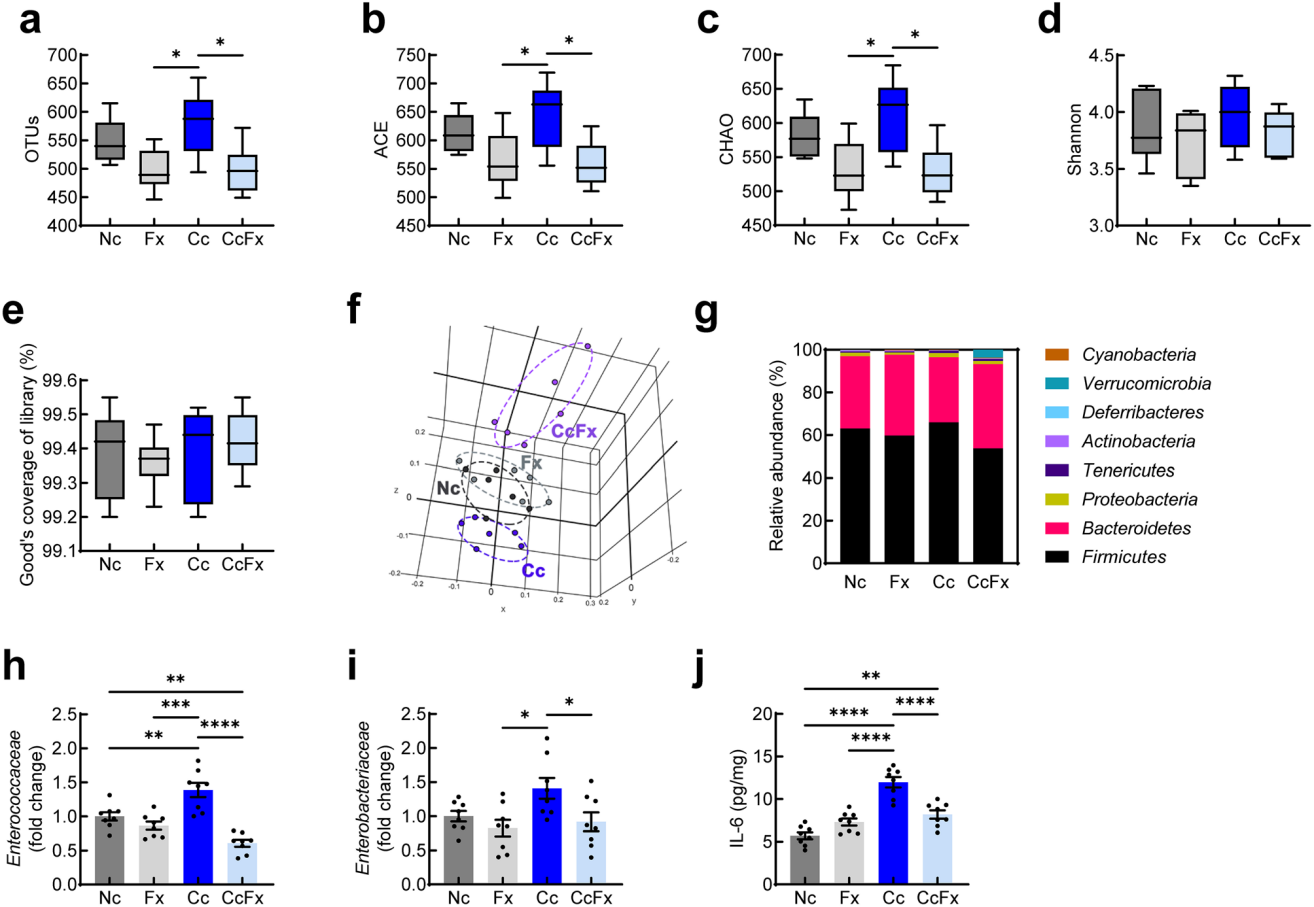
Effect of fluoxetine on colitis and gut microbiota composition in cefaclor-induced depressive mice. Alpha-diversity OTUs (**a**), ACE (**b**), CHAO (**c**), Shannon (**d**), Good coverage of library (**e**) indexes and beta-diversity (principal component analysis, PCoA) (**f**). Relative abundance of bacterial composition at the phylum level (**g**) assessed by pyrosequencing (n = 6/group). The relative abundance of *Enterococcaceae* (**h**) and *Enterobacteriaceae* (**i**) in the feces, analyzed by qPCR (n = 8/group). The effect of fluoxetine on the expression of IL-6 (**j**) in the colon (n = 8/group). Data are represented as mean ± S.E.M. Control mice, dark gray bar; mice subjected to fluoxetine alone, light gray bar; mice subjected to cefaclor, blue bar; mice subjected to cefaclor followed by fluoxetine, light blue bar. Statistical significance was calculated using a one-way ANOVA with post-hoc Tukey’s multiple comparisons tests (*p < 0.05, **p < 0.01, ***p < 0.001, ****p < 0.0001).

In the colon, 16S rRNA gene sequencing data showed that fluoxetine affected the cefaclor-induced gut dysbiosis and alteration of bacterial composition. There were significant differences in the OTUs, ACE, and CHAO indexes indicating gut microbial α-diversity, while there was no significant difference in the Shannon and Good’s coverage of library indexes [*F* (3, 20) = 4.947, *p* = 0.0099; Fig. 6a, F (3, 20) = 4.649, *p* = 0.0127; Fig. 6b, F (3, 20) = 5.329, *p* = 0.0073; Fig. 6c, F (3, 20) = 0.7191, *p* = 0.5522; Fig. 6d, and F (3, 20) = 0.2792, *p* = 0.8397; Fig. 6e].

At the phylum level, gut microbiota composition was different between the groups (Fig. 6f). Gut microbial β-diversity based on Bray–Curtis’s dissimilarity between samples showed that the overall gut microbiota community was different (PREMANOVA, *p* = 0.001; Fig. 6g). In line with the initial finding on culture plating (Fig. 1n,o), the qPCR analysis showed that the population of *Enterococcaceae* [*F* (3, 28) = 20.19, *p* < 0.0001; Fig. 6h] and *Enterobacteriaceae* [*F* (3, 28) = 4.197, *p* = 0.0143; Fig. 6i] increased in cefaclor-treated mice compared with control mice. Moreover, fluoxetine reduced the increased population of these bacteria in the gut microbiota. Fluoxetine decreased the elevated levels of IL-6, IL-1β, TNF-α, and myeloperoxidase in the colon of mice treated with cefaclor [*F* (3, 28) = 30.05, *p* < 0.0001; Fig. 6j, F (3, 28) = 14.07, *p* < 0.0001; Fig. S4a, *F* (3, 28) = 22.53, *p* < 0.0001; Fig. S4b, and *F* (3, 28) = 20.49, *p* < 0.0001; Fig. S4c].

## Discussion

There is increasing recognition of the adverse psychiatric effects of antibiotics in humans^5,11,31,32^. Of the antibiotics, ampicillin, ciprofloxacin, and azithromycin cause anxiety- and depression-like behaviors in mice^20,33,34^. Indeed, ampicillin, levofloxacin, and moxifloxacin mix have been reported to increase the risk of anxiety and depression in humans^35–37^. Antibiotic exposure induces gut dysbiosis and inflammation in mice^13,14^. It has been reported that antibiotics-induced gut microbiota dysbiosis such as an increase in *proteobacteria* may contribute to susceptibility to stress-related disorders in mice^38^. Probiotics treatment alleviated gut microbiota dysbiosis and inflammation, resulting in improvement in depression-like behaviors and cognitive impairment in antibiotics-exposed mice^12,16,39–42^. On the other hand, some studies have shown that antibiotics such as minocycline can have beneficial effects on depression by reducing inflammation and restoring the balance of the gut microbiota^43–45^. These studies suggest that antibiotics may reduce the diversity and abundance of beneficial bacteria and increase the growth of pathogenic bacteria in the gut, which can affect mood and cognition by producing harmful metabolites and inflammatory molecules that can reach the brain via the vagus nerve or the blood circulation.

In this study, oral administration of cefaclor induced anxiety- and depression-like behaviors, colitis, and gut microbiota dysbiosis in mice. Cefaclor decreased serotonin and BDNF levels in the hippocampus, whereas it increased kynurenine levels and *Ido1* mRNA expressions in the hippocampus. Cefaclor consistently modulated mRNA expression of *Htr1a*, *Htr1b*, and *Htr6* receptors, which are implicated in pathology of anxiety and depression, in the hippocampus.

Cefaclor altered gut microbiota composition, resulting in gut dysbiosis. Consistent with previous reports^26,46^, we found the increased number of *Enterococcaceae* and *Enterobacteriaceae* belong to cephalosporins-induced drug-resistant and conditionally pathogenic bacteria. The overgrowth of these bacteria can increase the risk for antibiotics-associated psychiatric disorders. Indeed, it has been reported that oral gavage of bacteria belonging to *Enterococcaceae* and *Enterobacteriaceae* induces anxiety- and depression-like behaviors in mice^20,25,47^.

Recent studies showed that subdiaphragmatic vagotomy blocked depression-like behaviors and reduced inflammation, abnormal composition of gut microbiota, and microbe-derived metabolites in mice^48,49^. These studies suggest that vagus nerve may block depression-like behaviors in rodents through the gut-microbiota-brain axis. However, more research is needed to elucidate the mechanisms of vagus nerve on depression and other psychiatric disorders. The vagus nerve plays an important role in communication with the central nervous system (CNS) along afferent and efferent pathways to transmit serotonin-involved signals from the gut to the brain^50,51^. Serotonin is a neurotransmitter that regulates the functions of CNS in anxiety and depression^52^. However, 95% of the body's serotonin is biosynthesized in the gut and 5-HT receptors are found in enterocytes and enteric neurons, affecting the change in 5-HT levels and 5-HT receptor expression in the brain^53^. Serotonin levels and 5-HT receptor expression is associated with the pathogenicity and treatment of depression^52,54^.

In the present study, vagotomy attenuated the anxiety- and depression-like behaviors induced by cefaclor in mice. Hippocampal BDNF, serotonin, *Htr4* mRNA, and *Htr6* mRNA expression levels were significantly associated with three variation factors including cefaclor, vagotomy, and interaction. The mRNA levels of kynurenine, and *Ido1* are significantly increased by cefaclor administration without vagotomy. On the other hand, pro-inflammatory cytokines such as IL-6, IL-1β, TNF-α are significantly increased by cefaclor administration with or without vagotomy. Cefaclor increases the levels of IL-6, IL-1β, and TNF-α in the plasma of mice with or without vagotomy. In the colon, the cefaclor-induced levels of IL-6, IL-1β, TNF-α, and myeloperoxidase were not different between sham-operated and vagotomized mice. These results suggest that cefaclor may cause neuroinflammation through gut dysbiosis-associated immune activation and its related bacteria and byproducts into the blood from the gastrointestinal tract.

One of the interesting results is that the reduction of hippocampal BDNF levels was prevented by vagotomy in mice treated with cefaclor. BDNF shows distinct signaling systems in the regulation of neuronal functions from serotonin in mood disorders^55^. Meanwhile BDNF also promotes the differentiation of 5-HT neurons and enhances serotonin receptor gene expression^56^. Conversely, serotonin-associated signals affect BDNF expression. In our cefaclor-induced anxiety and depression-like symptoms, even though there is a need to further investigation of the interaction between BDNF and serotonin signaling via the vagus nerve, vagotomy prevented the reduction of BDNF levels in the hippocampus of cefaclor-fed vagotomized mice compared with cefaclor-fed sham-operated mice. These results suggest that cefaclor may suppress BDNF and serotonin expression through gut microbiota-mediated blood circulation and vagus nerve pathways, resulting in the occurrence of anxiety and depression.

Vagotomy affected the gut microbial composition and diversity between groups. The relative abundance of *Firmicutes* decreased in cefaclor-fed vagotomized mice compared with the other groups. On the other hand, the relative abundance of *Proteobacteria* increased in cefaclor-fed vagotomized mice compared with the other groups. Vagotomy inhibited an increase in the number of *Enterococcaceae* and *Enterobacteriaceae* in cefaclor-fed vagotomized mice compared with cefaclor-fed sham-operated mice. The interaction between the vagus nerve and gut bacteria belong to *Enterococcaceae* and *Enterobacteriaceae* might contribute to the change of serotonin and BDNF levels in the hippocampus. Recent studies suggested that opportunistic pathogens such as *Enterococcus* and LPS may be associated with vagus nerve-mediated depression^48^. Thus, further studies on the mechanisms underlying how bacteria, at the species levels, belonging to *Enterococcaceae* and *Enterobacteriaceae* affect vagal afferents in anxiety and depression may give insight into this matter.

Furthermore, we investigated the effects of fluoxetine on cefaclor-induced depression in mice with or without celiac vagotomy. We observed that fluoxetine treatment improved the cefaclor-induced reduction of serotonin and BDNF levels as well as the expression of pro-inflammatory cytokines, resulting in decrease anxiety- and depression-like behaviors in mice. Fluoxetine restored the decreased expression of *Htr1a* mRNA, and the increased expression of *Htr1b* mRNA and *Htr6* mRNA in the hippocampus of mice treated with cefaclor. The expression of *Htr4* mRNA increased in the hippocampus of mice treated with fluoxetine. The 5-HT4 receptor expressed in the gastrointestinal tract might involve in the modulation of mood disorders in the hippocampus^57,58^. These findings support that the therapeutic effects of fluoxetine are associated with restoring 5-HT signaling, modulating the immune response, increasing BDNF, and activating vagus nerve-gut-brain signaling^30,59–62^. Fluoxetine affected gut microbiota composition and showed antibacterial effects on cefaclor-resistant gut bacteria^63^. Gut microbial diversity and the increased abundance of *Enterococcaceae* and *Enterobacteriaceae* were attenuated by fluoxetine^64^. These results suggest that fluoxetine, an antidepressant medication, has been shown to affect the gut microbiota and may contribute to its therapeutic effects in depression accompanied by gut dysbiosis.

In conclusion, cefaclor caused gut dysbiosis, overgrowing pathogenic gut bacteria such as *Enterococcaceae* and *Enterobacteriaceae,* and anxiety- and depression-like behaviors in mice. Consistent with the behavior changes, cefaclor reduced BDNF, serotonin levels, and 5-HT1A receptor mRNA expression in the hippocampus. Vagotomy attenuated the changes in cefaclor-induced depressive symptoms. Treatment of fluoxetine alleviated cefaclor-induced anxiety- and depression-like symptoms and gut dysbiosis in mice. These finding suggest that cefaclor-induced gut dysbiosis may cause anxiety and depression through the microbiota-gut-blood–brain and the microbiota-gut-vagus nerve-brain pathway. Finally, targeting antibiotic-resistant pathogenic bacteria may be a promising therapeutic strategy for the treatment of anxiety and depression.

## Materials and methods

### Animal

C57BL/6J mice (male, 5 weeks old) were purchased from DBL Co., Ltd. (Eumseong, Korea). Mice were housed in groups of 6–8/cage in standard cages and were under controlled conditions (temperature 20 °C–21 °C, and humidity 55–60%) on a 12 h light/dark cycle with access to food and water ad libitum. Mice were habituated for a week before experiments. All animal procedures were ethically approved by the Ethics Committee of Animal Experiments in Kyung Hee university IACUC (approval number: KHUAPS(SE)-21-525).

### Cefaclor and fluoxetine administration

To prepare the antibiotics-induced depression model, mice were allocated randomly in each group (n = 6/group). Mice were administrated orally with cefaclor (200 mg/kg/day, dissolved in 0.2 mL saline) once a day for 5 days. Control groups were treated with vehicle (0.2 mL saline) instead of cefaclor. The doses of cefaclor used in the study were established according to their clinical doses^65^ and equivalent dose calculation based on body surface area^66^. Cefaclor (PHR1283) was purchased from Sigma-Aldrich (St. Louis, MO).

To examine the antidepressant effects of fluoxetine on cefaclor-induced anxiety- and depression-like behaviors in mice, mice were allocated randomly to each group (n = 8/group). fluoxetine (20 mg/kg/day, dissolved in 0.2 mL saline) was orally administrated once a day for 5 days^67^. Control groups was treated with vehicle (0.2 mL saline) instead of fluoxetine. Fluoxetine (PHR1394) was purchased from Sigma-Aldrich.

### Celiac vagotomy

Celiac vagotomy was performed as previously reported^68^. Mice were anesthetized with 1.5–4% isoflurane. An incision was made slightly to the right side of the abdominal midline. The liver was moved gently using a sterile cotton swab to expose the stomach and esophagus. The celiac branch of the vagus nerve was resected. The stomach and esophagus were exposed in the sham operation, whereas the vagus nerve was not resected. The incision was closed using a non-absorbable suture and treated with povidone-iodine to prevent infection. The mice were kept in a clean cage for 5 days after surgery. Celiac vagotomy was validated by measuring fecal pellet parameters. The total number of the excreted pellets, fecal water content, and fecal length were determined^69^. Validated mice were used in the next experiments.

### Behavioral tasks

#### Open field test

The OFT was used to evaluated the effects on anxiety-related behaviors according to Seibenhener, M.L. et al.^70^. In the test, mice were placed in an open arena divided into a central area and a peripheral area of a chamber (40 cm × 40 cm). The animal’s movement and behavior were recorded for 10 min. During the test, the distance traveled, the time spent in the center, and the velocity were measured using the EthoVision XT software.

#### Elevated plus maze

The EPM was used to evaluate anxiety-related behaviors, as previously reported^25^. It consists of a plus-shaped platform with two open arms and two closed arms (30 × 6 × 20 cm walls) that were elevated at a height of 40 cm above the bottom. Mice were placed on the central platform facing one of the open arms. The animal’s behavior was recorded for 5 min. The percentage of time spent in open arms and the number of entries into open arms were measured and corrected by total time or entries in open plus closed arms, respectively.

#### Tail suspension test

The TST was used to evaluate depression-related behaviors, as previously reported^71^ with minor modifications^72^. During the test, mice were suspended by its tail using a clip with their body positioned vertically. The amount of immobile time was recorded for 6 min. The percentage of total immobility time was quantified.

#### Forced swim test

The FST was used to evaluate depression-related behaviors^73^. During the test, mice were individually placed in a transparent cylinder (15 cm in diameter and 30 cm in height) with water to a depth of 15 cm (24–25 °C). The amount of immobile time was recorded for 6 min. The percentage of total immobility time was quantified.

### Quantitative real-time polymerase chain reaction (qPCR) analysis

Total RNA was extracted from hippocampus tissue by using Qiagen RNeasy mini kit (cat. 74,106, Qiagen, Hilgen, Germany). The isolated 2 μg of RNA was reverse-transcribed using a PrimeScript cDNA synthesis kit (cat. 6110A, Takara, Shiga, Japan). During the PCR amplification, TB Green Premix Ex Taq II (cat. RR820A, Takara) were used and the PCR reaction was performed using the Rotor-Gene Q 5plex Platform (cat. 9,001,570, Qiagen). Gene expression of indoleamine 2,3-dioxygenase 1 (IDO1), 5-HT_1A_, 5-HT_1B_, 5-HT_4_, 5-HT_6_, 5-HT_7_, and glyceraldehyde 3-phosphate dehydrogenase (GAPDH) were analyzed^58,74–76^. GAPDH was used as a housekeeping gene to normalize the relative mRNA expression levels.

For fecal bacterial analysis, fecal bacterial DNA was extracted from colon contents by using the QIAamp Power Fecal Pro DNA kit (Cat. 51,804, Qiagen). Primer sequences of *Enterococcaceae* spp., *Enterobacteriaceae* spp., and 16S rRNA were used as previously reported^77,78^. 16S rRNA was used as a housekeeping gene to normalize the relative mRNA expression levels in the feces. The primer sequences used for qPCR amplification are shown in Table S1. Reaction conditions were employed as follows: 95°C for 30 s; 95°C for 15 s, 60°C for 20 s, 72°C for 20 s, and 40 cycles.

### Enzyme-linked immunosorbent assay (ELISA)

Protein lysate was extracted from hippocampus and colon tissues by using RIPA buffer (150mM sodium chloride, 1% Triton X-100, 1% sodium deoxycholate, 0.1% SDS, 50mM Tris–HCl, 2mM EDTA, pH7.5) containing protease inhibitor cocktail (Roche) and phosphatase inhibitor cocktail (Roche). The concentration of protein was determined by Bradford assay. For plasma collection, whole blood was collected into EDTA-treated tubes by cardiac puncture. Cells were removed from the plasma by centrifugation for 20 min at 4000 rpm. In the hippocampus, the colon, and the plasma, pro-inflammatory cytokines levels were detected using TNF-α (DY410, B&D system), IL-1β (DY401, B&D system), IL-6 (DY406, B&D system) ELISA kits. Hippocampal serotonin, kynurenine, and brain-derived neurotrophic factor (BDNF) levels were detected by serotonin (SER39-K01, Eagle Biosciences), kynurenine (BA E-2200, Eagle Biosciences), and BDNF (DY248, B&D system) ELISA kit. Colonic myeloperoxidase expression was detected by MPO (DY3667, R&D system) ELISA kit. ELISA was performed according to the manufacturer’s instructions.

### Determination of intestinal microbiota

The fecal pellets were diluted 10 times (w/v) with Gifu Anaerobic Medium (GAM) broth under anaerobic conditions. The diluted samples (100 μL) were plated onto the surface of culture mediums, including mEnterococcus (mE) agar plates, Deoxycholate-Hydrogen Sulfide-Lactose (DHL) agar plates, and GAM agar plates with or without cefaclor (50 μg/mL). The plates were incubated at 37 °C for 48 h. The number of colonies on the plate was counted. The colony forming unit per fecal weight was calculated.

### Microbial DNA extraction and 16S rRNA gene sequencing

Mice were euthanized and colon contents were collected into autoclaved tubes and stored at − 80 °C until further analysis. Fecal bacterial DNA was extracted using the QIAamp Power Fecal Pro DNA kit (Cat. 51,804, Qiagen) and amplified using barcoded primers targeting the bacterial 16S rRNA V3-V4 gene region. Amplicon sequencing was performed using Illumina iSeq 100 (San Diego, CA). The fecal microbiota was analyzed using the EzBioCloud database and 16S microbiome pipeline (https://www.EZbiocloud.net). Sequenced reads were stored in the NCBI's short read archive (accession number PRJNA895548).

### Statistics

All data are presented as mean ± standard error (S.E.M). The Shapiro–Wilk test was used to assess data normal distribution. To analyze statistical significances, a two-tailed unpaired t-test on two groups of data with normal distributions, and one-way ANOVA or two-way ANOVA with Tukey’s multiple post hoc were used in multiple group analysis. To analyze nonparametric data, the Kruskal–Wallis test with Dunn’s multiple comparisons test was used. The graphical representations were carried out with GraphPad Prism 10 (San Diego, USA).

### Supplementary Information

Supplementary Information.

## Data Availability

All data supporting our findings can be found in the main paper or supplementary files. Sequencing data are available via the NCBI's short read archive (Accession Number PRJNA895548).

## Abbreviations

ANOVA: One-way analysis of variance
BDNF: Brain-derived neurotrophic factor
Cc: Cefaclor
CFU: Colony forming unit
DHL: Deoxycholate-hydrogen sulfide-lactose
ELISA: Enzyme-linked immunosorbent assay
EPM: Elevated plus maze
FST: Forced swim test
Fx: Fluoxetine
GAM: General anaerobic medium
GABA: γ-Aminobutyric acid
gDNA: Genomic DNA
IL: Interleukin
mE: Membrane filter Enterococcus selective medium
RIPA: Radioimmunoprecipitation assay
SDS–PAGE: Sodium dodecyl sulfate–polyacrylamide gel electrophoresis
TNF: Tumor necrosis factor
TST: Tail suspension test
OFT: Open field test
Vx: Vagotomy
