# Supplementary material for: Cefaclor causes vagus nerve-mediated depression-like symptoms with gut dysbiosis in mice
Source: Sci Rep. 2023 Sep 19;13:15529. doi: 10.1038/s41598-023-42690-1 (PMC10509198; doi:10.1038/s41598-023-42690-1)
Supplement: Supplementary file 1 — Supplementary Information. [file 41598_2023_42690_MOESM1_ESM.docx]

**[Supplementary Material]**

**Cefaclor causes vagus nerve-mediated depression-like symptoms with gut dysbiosis in mice**

**
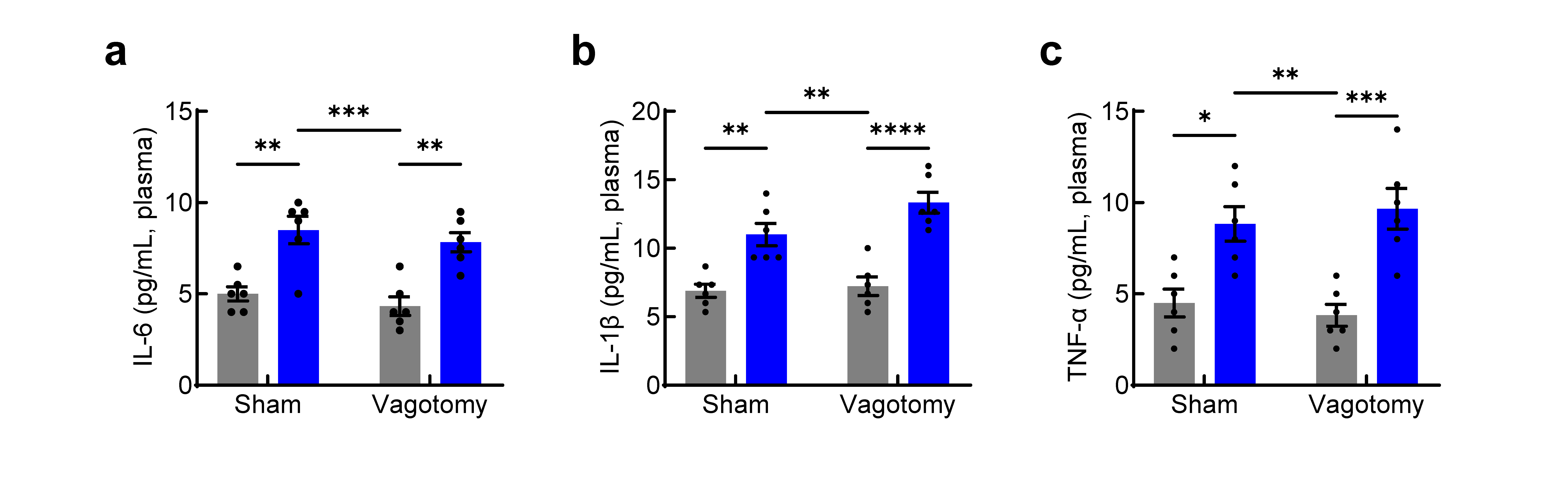
**

**Figure S1.** **Effect of orally administered cefaclor on pro-inflammatory cytokine expression in the plasma of vagotomized mice.** The level of IL-6 (a), IL-1β (b), and TNF-α (c) in the plasma. Control group, dark gray bar; cefaclor-treated group, blue bar. Data are represented as mean ± S.E.M (n = 6/group). Statistical significance was calculated using a two-way ANOVA with post-hoc Tukey’s multiple comparisons tests (*P < 0.05, **P < 0.01, ***P < 0.001, ****P < 0.0001).


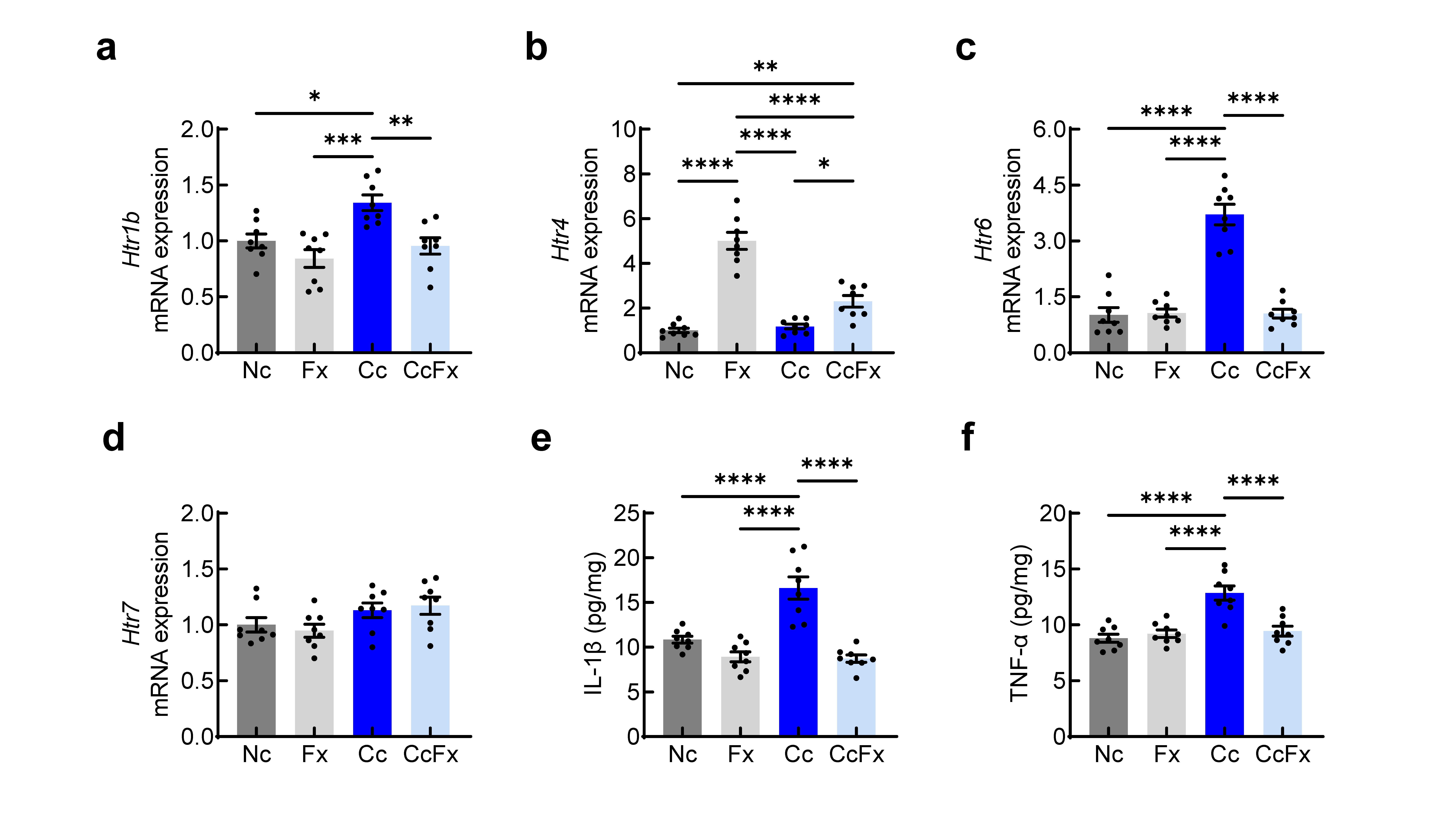


**Figure S2. Effect of fluoxetine on the expression of serotonin, 5-HT receptor, and pro-inflammatory cytokine in the hippocampus of cefaclor-induced depressive mice.** The effect of fluoxetine on the expression of *Htr1b* (a), *Htr4* (b), *Htr6* (c), and *Htr7* (d) mRNA in the hippocampus. The expression of IL-1β (e), TNF-α (f) in the hippocampus. Control mice, dark gray bar; mice subjected to fluoxetine alone, light gray bar; mice subjected to cefaclor, blue bar; mice subjected to cefaclor followed by fluoxetine, light blue bar. Data are represented as mean ± S.E.M (n = 8/group). Statistical significance was calculated using a one-way ANOVA with post-hoc Tukey’s multiple comparisons tests (*P < 0.05, **P < 0.01, ***P < 0.001, ****P < 0.0001).


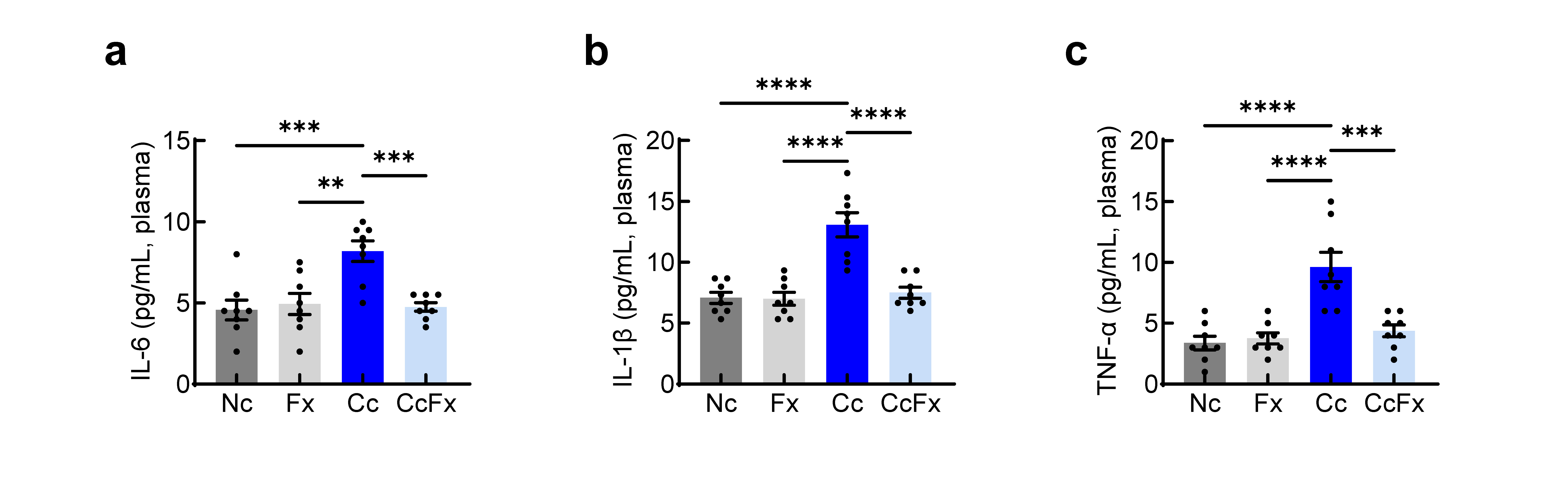


**Figure S3.** **Effect of fluoxetine on pro-inflammatory cytokine expression in the plasma of cefaclor-induced depressive mice.** The level of IL-6 (a), IL-1β (b), and TNF-α (c) in the plasma. Control mice, dark gray bar; mice subjected to fluoxetine alone, light gray bar; mice subjected to cefaclor, blue bar; mice subjected to cefaclor followed by fluoxetine, light blue bar. Data are represented as mean ± S.E.M (n = 8/group). Statistical significance was calculated using a one-way ANOVA with post-hoc Tukey’s multiple comparisons tests (*P < 0.05, **P < 0.01, ***P < 0.001, ****P < 0.0001).

**
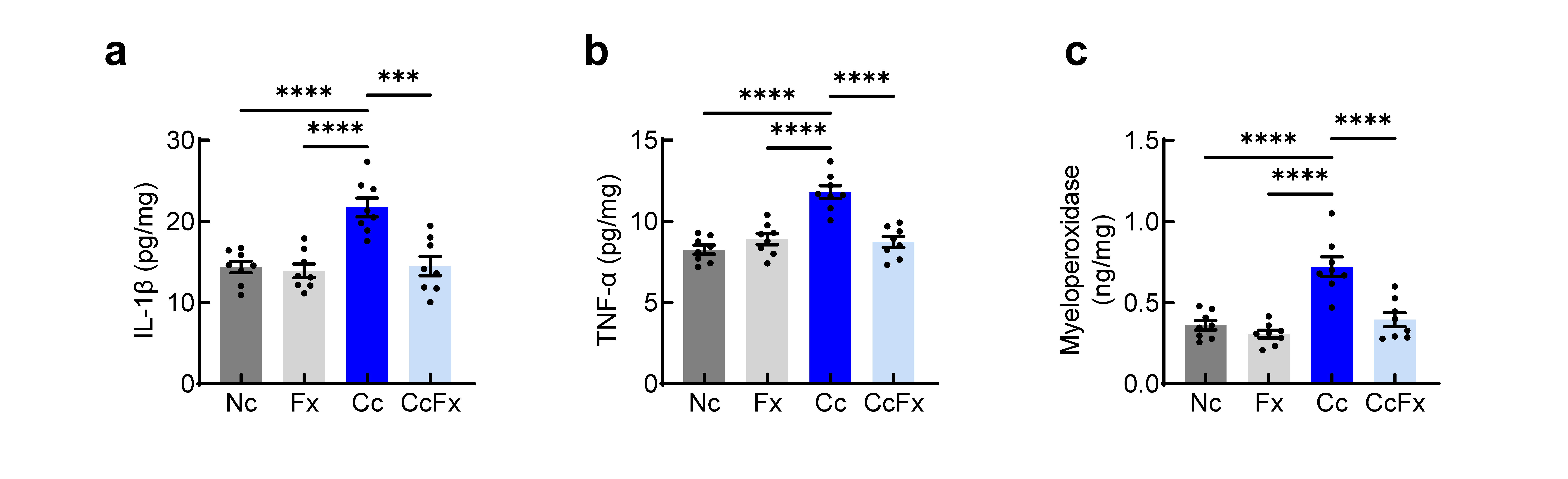
**

**Figure S4. Effect of fluoxetine on colitis and in cefaclor-induced depressive mice.** The effect of fluoxetine on the expression of IL-1β (a), TNF-α (b), and myeloperoxidase (c) in the colon. Control mice, dark gray bar; mice subjected to fluoxetine alone, light gray bar; mice subjected to cefaclor, blue bar; mice subjected to cefaclor followed by fluoxetine, light blue bar. Data are represented as mean ± S.E.M (n = 8/group). Statistical significance was calculated using a one-way ANOVA with post-hoc Tukey’s multiple comparisons tests (*P < 0.05, **P < 0.01, ***P < 0.001, ****P < 0.0001).

**Table S1.** **The primer sequences used for qPCR**

| **Table S1. The primer sequences used for qPCR** | | |
| --- | --- | --- |
| 5-HT_1A_ | Forward | 5'-CCGTGAGAGGAAGACAGTGAAGA-3' |
|  | Reverse | 5'-GGTTGAGCAGGGAGTTGGAGTAG-3' |
| 5-HT_1B_ | Forward | 5'-ACATCCTCGGTCACCTCCATTA-3' |
|  | Reverse | 5'-CCCTAGCGGCCATGAGTTTC-3' |
| 5-HT_4_ | Forward | 5'-TCGAGGCATTCCTCGATTCA-3' |
|  | Reverse | 5'-TAACACCTGGCCGAAACGTT-3' |
| 5-HT_6_ | Forward | 5'-CCATTCTCAACCTCTGCCTCAT-3' |
|  | Reverse | 5'-GCAAGATCCTGCAGTAGGTGAA-3' |
| 5-HT_7_ | Forward | 5'-GGCTACACGATCTACTCCACCG-3' |
|  | Reverse | 5'-CGCACACTCTTCCACCTCCTTC-3' |
| IDO1 | Forward | 5'-CAAAGCAATCCCCACTGTATCC-3' |
|  | Reverse | 5'-ACAAAGTCACGCATCCTCTTAAA-3' |
| GAPDH | Forward | 5'-TGCAGTGGCAAAGTGGAGAT-3' |
|  | Reverse | 5'-TTTGCCGTGAGTGGAGTCATA-3' |
| *Enterococcaceae* | Forward | 5'-CCCTTATTGTTAGTTGCCATCATT-3' |
|  | Reverse | 5'-ACTCGTTGTACTTCCCATTGT-3' |
| *Enterobacteriaceae* | Forward | 5'-GTGCCAGCMGCCGCGGTAA-3' |
|  | Reverse | 5'-GCCTCAAGGGCACAACCTCCAAG-3' |
| 16S rRNA | Forward | 5'-TCGTCGGCAGCGTCAGATGTGT ATAAGAGACAGGTGCCAGCMGCCGCGGTAA-3' |
|  | Reverse | 5'-GTCTCGTGGGCTCGGAGATGT GTATAAGAGACAGGGACTACHV GGGTWTCTAAT-3' |
